# Initial Site of Metastasis Influences Prognosis in Pancreatic Ductal Adenocarcinoma

**DOI:** 10.1002/cam4.71760

**Published:** 2026-03-26

**Authors:** Eleanor Mancheski, David Baek, Wilbur Bowne, Harish Lavu, Charles J. Yeo, Avinoam Nevler

**Affiliations:** ^1^ Sidney Kimmel Medical College Thomas Jefferson University Philadelphia Pennsylvania USA; ^2^ Jefferson Pancreatic, Biliary and Related Cancer Center, Sidney Kimmel Comprehensive Cancer Center Thomas Jefferson University Philadelphia Pennsylvania USA

**Keywords:** metastasis, oncologic outcomes, pancreatic ductal adenocarcinoma, PDAC

## Abstract

**Introduction:**

Metastatic pancreatic ductal adenocarcinoma (mPDAC) is a highly aggressive malignancy. Prior studies suggest that the initial site of metastasis may impact prognosis. This study investigates whether overall survival in mPDAC patients differs between patients first presenting with lung metastases versus those presenting with liver metastases.

**Methods:**

This retrospective analysis utilized the multi‐institutional TriNetX database, identifying patients with histologically diagnosed PDAC who initially presented with either liver or lung metastases. Demographic, histologic, and outcome data were collected and analyzed. Patients were matched 1:1 using a nearest neighbor propensity score (PS) algorithm, and Kaplan–Meier survival analyses were performed. Cox regression was also used to further validate the results of the PS matching.

**Results:**

A total of 6256 patients were identified, including 5390 patients presenting with liver and 866 patients presenting with lung metastases at diagnosis. The mean age was 66.6 ± 10.5, the male‐to‐female ratio was 54%:46%, the mean carbohydrate‐antigen (CA) 19‐9 level was 1309 ± 2078 ng/mL, the mean carcinoembryonic‐antigen (CEA) level was 117 ± 1044 U/mL. Propensity score matching yielded 848 matched pairs. Median survival from time of metastatic diagnosis was significantly longer for patients with lung metastases compared to liver (377 vs. 195 days, *p* < 0.0001). Cox regression identified several factors associated with increased risk of death: older age, obesity, malnutrition, and elevated CEA or CA 19‐9. Additionally, initial lung metastases were associated with decreased risk of death (HR = 0.61, *p* < 0.0001).

**Conclusion:**

Initial presentation with lung metastases appears to be associated with improved survival outcomes as compared to initial presentation with liver metastases in patients with mPDAC.

## 1 Introduction

Pancreatic ductal adenocarcinoma (PDAC) is one of the most common and highly aggressive malignancies of the pancreas [1]. It has now become the third leading cause of cancer death in United States with an estimated 67,440 new cases and 51,980 deaths this year, amounting to a 5‐year overall survival [OS] of 13% [2]. Unfortunately, most patients are identified at an advanced disease stage with distant metastases, for which the 5‐year OS is only 3% [3].

The common metastatic sites in order are liver, lung, peritoneum, and distant lymph nodes, with liver being the most common site and with the poorest prognosis [4]. The mechanisms of regulating the fashion through which PDAC metastasized to different organ sites have only recently begun to be understood [5, 6, 7]. Investigations into each metastatic site's unique tumor microenvironment are still ongoing [8, 9, 10], after small‐scale studies have indicated that the initial metastatic site in other cancers may impact prognosis [11, 12, 13].

Prior studies have demonstrated site‐dependent patient survival differences in various cancers, such as lung, breast, and colorectal adenocarcinomas [11, 12, 13]. For example, Cavallaro et al. noted an improved survival in colorectal cancer patients initially presenting with lung metastases compared to those who first presented with liver metastases [13]. Similar small‐scale studies have suggested that this association also exists in PDAC patients who first present with lung metastases as compared to those with liver metastases [14, 15]. There have been several proposed hypotheses regarding the mechanism of this observed association, including inherent differences in the immunogenicity of the tumor microenvironment (TME) of the different metastatic sites [16], and baseline site‐specific tropism driven by the genetic mutational landscape of the primary or circulating tumor cells, which may contribute to such differences in prognosis [17, 18, 19].

In this study, we aimed to use a larger‐scale multi‐institutional database from multiple countries to create a global and more generalizable assessment of the impact of the initial presenting metastatic site on the long‐term prognosis of PDAC patients.

## 2 Methods

### 2.1 Data Source and Setting

This was a retrospective study using TriNetX, a global federated electronic health record (EHR) research network [20]. TriNetX aggregates data from healthcare organizations (HCOs), including academic medical centers, community hospitals, and outpatient clinics. For this study, we used retrospective data from 109 HCOs within the research network. All data are mapped to standardized terminologies: diagnoses to ICD‐10‐CM; procedures to ICD‐10‐PCS, ICD‐9‐CM, or current procedural terminology (CPT); laboratory tests to logical observation identifiers names and codes (LOINC). Histologic annotations of cancer type were also provided for the primary tumor.

### 2.2 Study Population

The TriNetX platform is a multi‐institutional research network. It includes electronic health records from over 160 million patients. To ensure compliance with health insurance portability and accountability act (HIPAA) regulations, all protected health information (PHI) is removed. For this study, the TriNetX database was queried for ICD‐10 codes to identify patient populations and only cases with annotated pathology of adenocarcinoma were included.

We created two cohorts: (1) PDAC patients with initial liver only metastases (Initial liver cohort; Table S1) and (2) PDAC patients with initial lung only metastases (Initial lung cohort; Table S2). All data were collected from TriNetX on September 15, 2025. The liver‐first cohort was identified using ICD‐10‐CM codes (see Supporting Information section) and filtered to include only PDAC patients. Patients with endocrine pancreatic malignancies were excluded. Additionally, patients who had any record of a secondary malignant neoplasm of the lung (ICD‐10‐CM C78.0) prior to liver metastases were excluded. This query yielded 5390 patients.

The initial lung cohort was identified similarly, using ICD‐10‐CM codes and the same filters to ensure inclusion of only PDAC patients. Patients with prior secondary malignant neoplasm of the liver or intrahepatic bile ducts were excluded. This query yielded 866 patients.

### 2.3 Statistical Analysis

For statistical testing in the TriNetX analysis, categorical variables were compared using the Chi‐squared test, and numerical variables were compared using the student's *t*‐test.

Laboratory values were made into categorical variables. Carcinoembryonic antigen in serum was categorized into 0–5, 5–10, and > 10 ng/mL. Carbohydrate antigen 19‐9 in serum or plasma categories were 0–37, 37–500, 500–1000, and > 1000 Units/mL.

Propensity score matching was performed based on age on presentation, gender, nutritional status (ICD‐10 diagnoses of obesity or malnutrition), and serum biomarker levels (CA 19‐9 and CEA) to create comparable cohorts using a 1:1, nearest neighbor methodology. A standardized mean difference of less than 0.25 was considered a measure of appropriate matching.

Following propensity score matching, a Kaplan–Meier analysis was conducted to estimate overall survival, and a log‐rank test was used to compare survival between the two groups. A hazard ratio was calculated to quantify the relative risk of death. The index event for both cohorts was the time of diagnosis of metastatic disease, and the primary outcome was death. To assess the impact of various risk factors on survival, a Cox proportional hazards model was applied, adjusting for relevant covariates. Confidence intervals (CI) reported were 95%. *p* values below 0.05 were considered statistically significant.

### 2.4 Ethics

This study was performed at Thomas Jefferson University Hospital (TJUH) and approved by the local institutional review board (Control #iRISID‐2024‐0940).

## 3 Results

### 3.1 Demographic and Clinical Characteristics

A total of 6256 PDAC patients presenting with liver first or lung first metastases were identified in the TriNetX research network. Initial liver metastases were observed in 5390 patients and initial lung metastases in 866 patients. Descriptive statistics of the demographic and clinical characteristics are shown in Table 1. The mean age at diagnosis was 66.2 years for patients with liver metastases and 69.0 years for those with lung metastases (*p* < 0.001). Approximately 51.7% of patients with liver metastases were male, compared to only 42.8% in the lung metastases group (*p* < 0.001). Ten patients in the lung cohort (1%) and 24 patients in the liver cohort had a CPT code for surgical procedures on the pancreas. Marked differences in nutritional status were also noted between the two cohorts. Malnutrition was diagnosed in 4.5% of the liver cohort and 2.9% of the lung cohort (*p* = 0.03). A total of 1005 patients (16.1%) had recorded CEA levels prior to presentation. Similarly, 1645 patients (26.3%) had CA 19–9 levels recorded prior to presentation. Of these available data, patients presenting initially with lung metastasis had lower mean levels of CEA (17.4 ± 43.2 vs. 132.6 ± 1118.9, *p* = 0.04) and CA 19‐9 (953.8 ± 1781.4 vs. 1372.2 ± 1118.9, *p* < 0.001).

**TABLE 1:** Demographic characteristics.

	PDAC patients with initial liver metastases (*N* = 5390)	PDAC patients with initial lung metastases (*N* = 866)	*p*
Age at diagnosis (±SD)	66.2 ± 10.6	69.0 ± 9.8	< 0.001
Gender			< 0.001
Male	2787 (51.7%)	371 (42.8%)	
Female	2255 (41.8%)	449 (51.9%)	
Missing	348 (6.5%)	46 (5.3%)	
Race			0.02
White	3766 (69.9%)	645 (74.5%)	
Black or African American	818 (15.2%)	100 (11.5%)	
Hispanic or Latino	266 (4.9%)	42 (4.9%)	
Other/Unknown	540 (10.0%)	79 (9.1%)	
Overweight and obesity	197 (3.7%)	26 (3.0%)	0.32
Malnutrition	242 (4.5%)	25 (2.9%)	0.03
CEA (ng/mL)			0.04
0–5	388 (7.2%)	70 (8.1%)	
5–10	166 (3.1%)	29 (3.3%)	
> 10	349 (6.5%)	37 (4.3%)	
CA 19‐9 (U/mL)			< 0.001
< 37	294 (5.5%)	77 (8.9%)	
37–500	509 (9.4%)	92 (10.6%)	
500–1000	183 (3.4%)	22 (2.5%)	
> 1000	663 (12.3%)	73 (8.4%)	

Abbreviations: CA 19‐9, carbohydrate antigen 19‐9; CEA, carcinoembryonic antigen; PDAC, pancreatic ductal adenocarcinoma.

### 3.2 Survival Outcomes

A total of 5265 out of the 5390 patients (97.7%) from the liver metastasis cohort and 853 out of 866 patients (98.5%) from the lung metastasis cohort were available for survival analysis. A total of 138 patients were excluded during the survival analysis due to meeting the index event more than 20 years ago or capturing the event of death along with presentation. Of the final cohorts combined for survival analysis (*n* = 6118), the mean and median survival times were 353 ± 4.9and 213 ± 5.5 days, respectively. The 6‐month survival rate was 54.1% ± 0.6%, 1‐year survival rate was 33.9% ± 0.6%, and the 2‐year survival rate was 17.5% ± 0.5%.

**FIGURE 1:**
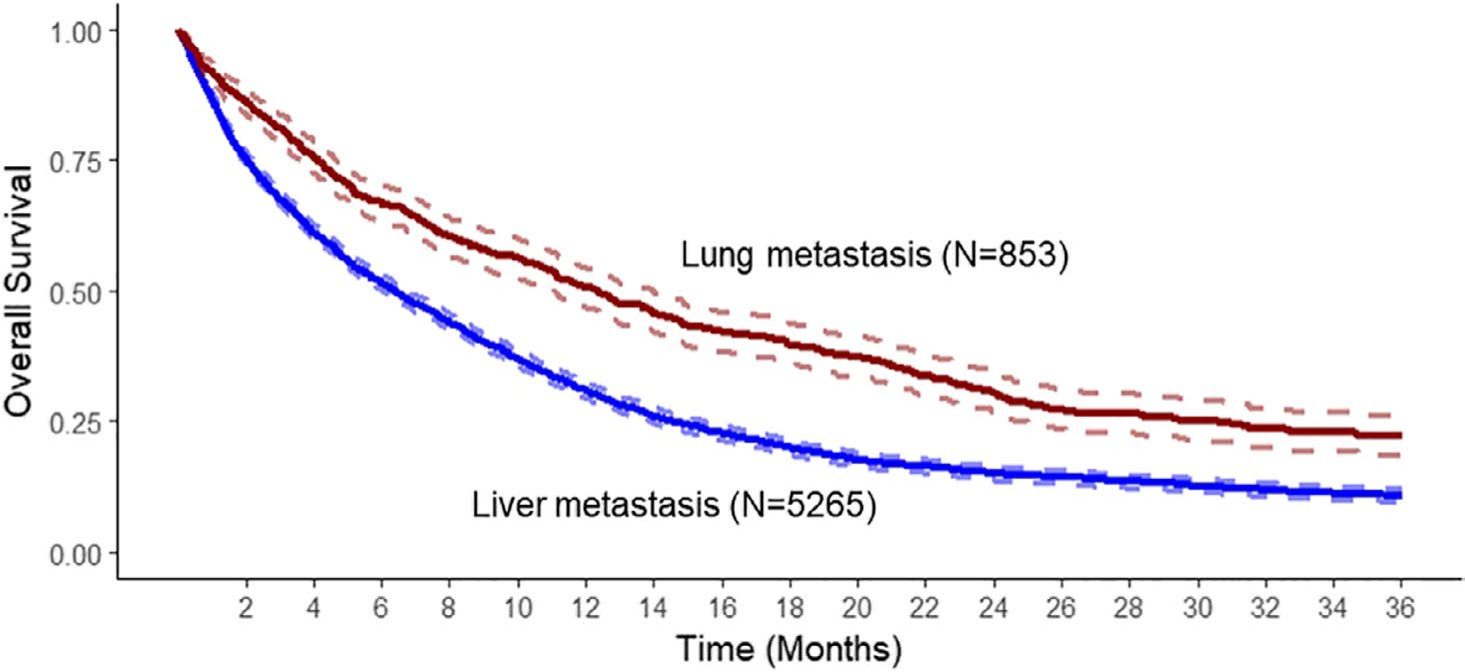
Kaplan–Meier analysis of overall survival in metastatic PDAC patients initially presenting with liver only or lung only metastases. Patients presenting with lung metastasis showed significantly better overall survival (12.4 vs. 6.4 months, *p* < 0.0001).

Patients presenting in the liver metastases cohort had a shorter overall survival as compared to patients first presenting with lung metastases (195 vs. 377 days, *p* < 0.0001. HR = 0.61, 95% CI 0.56–0.67) as seen in Figure 1.

### 3.3 Propensity Scoring Matched Survival Analysis

A total of 1696 patients (848 matched pairs) were identified after controlling for age at metastatic presentation, sex, race, nutritional status, CEA, and CA 19‐9 values. Both groups were similar in terms of age, sex, racial distribution, and presentation, as seen in Table 2.

**TABLE 2:** Propensity‐score matched cohort after 1:1 nearest neighbor matching for significant demographic and clinical features (*N* = 670 patient pairs).

	Matched PDAC patients with initial liver metastases (liver = 848)	Matched PDAC patients with initial lung metastases (lung = 848)	*p*
Age at diagnosis (± years SD)	69.1 ± 9.7	69.0 ± 9.8	0.78
Sex			0.92
Male	379 (44.7%)	371 (43.8%)	
Female	437 (51.5%)	444 (52.4%)	
Unknown	32 (3.8%)	33 (3.8%)	
Race			0.76
White	650 (76.7%)	642 (75.7%)	
Black or African American	94 (11.1%)	100 (11.8%)	
Hispanic or Latino	47 (5.5%)	41 (4.8%)	
Other/Unknown	57 (6.7%)	65 (7.7%)	
Overweight or obesity	21 (2.5%)	26 (3.1%)	0.46
Malnutrition	23 (2.7%)	25 (2.9%)	0.77
CEA (ng/mL)			0.96
0–5	61 (7.2%)	68 (8.0%)	
5–10	24 (2.8%)	29 (3.4%)	
> 10	31 (3.7%)	37 (4.4%)	
CA 19‐9 (units/mL)			0.74
< 37	69 (8.1%)	72 (8.5%)	
37–500	80 (9.4%)	92 (10.8%)	
500–1000	26 (3.1%)	22 (2.6%)	
> 1000	77 (9.1%)	73 (8.6%)	

Abbreviations: CA 19‐9, carbohydrate antigen 19‐9; CEA, carcinoembryonic antigen; PDAC, pancreatic ductal adenocarcinoma.

**FIGURE 2:**
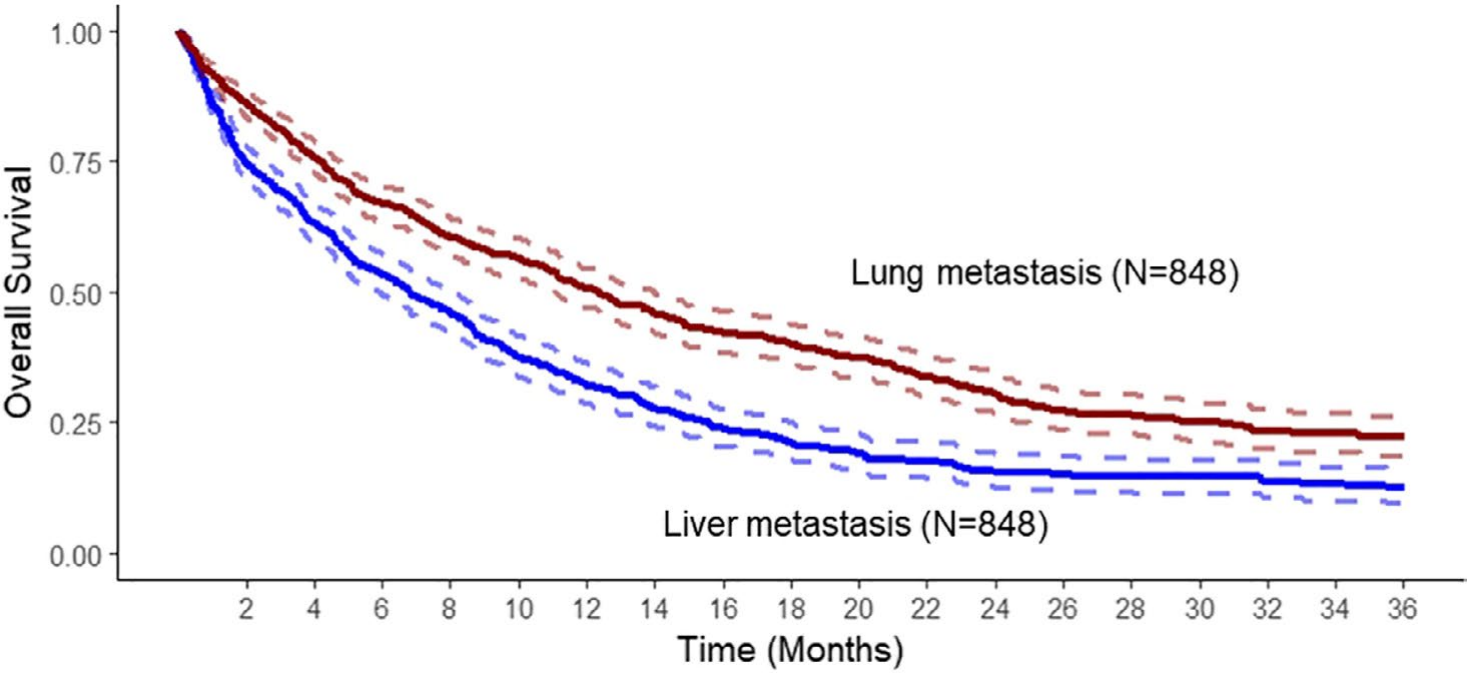
Kaplan–Meier analysis of overall survival in propensity‐score matched metastatic PDAC patients (*n* = 848 patient pairs) initially presenting with liver or lung metastases. Patients presenting with lung metastasis showed significantly better overall survival (6.8 vs. 12.4 months, *p* < 0.0001).

In this propensity score matching analysis, patients initially presenting with liver metastases displayed shorter median survival as compared to patients with lung metastases at presentation (208 vs. 377 days, *p* < 0.0001. HR = 0.64, 95% CI 0.56–0.72), as shown in Figure 2.

### 3.4 Cox Multi‐Hazard Regression Analysis

In order to investigate the prognostic impact of various risk factors, a Cox regression was employed (see Table 3). The analysis found that patients presenting with lung metastases experienced better survival as compared to patients in the liver metastases cohort (HR: 0.61, 95% CI: 0.56–0.68, *p* < 0.0001). Additionally, other key prognostic factors included age at index (HR: 1.01/year, 95% CI: 1.01–1.02, *p* < 0.0001), White race (HR: 0.88, 95% CI: 0.80–0.98, *p* = 0.014), overweight and obesity (HR: 1.18, 95% CI: 1.01–1.39, *p* = 0.044), and malnutrition (HR: 1.56, 95% CI: 1.34–1.82, *p* < 0.0001). Increased cancer biomarkers CEA (HR: 1.21, 95% CI: 1.06–1.39, *p* = 0.006) and CA 19‐9 (HR: 1.184, 95% CI: 1.07–1.31, *p* = 0.001) were also found to be highly associated with worse survival.

**TABLE 3:** Cox multi‐hazard regression model of overall survival.

Covariate	Hazard ratio (95% CI)	*p* > |*z*|
Lung mets vs. liver mets.	0.61 (0.56–0.68)	< 0.0001
Age at index (risk/year)	1.01 (1.01–1.02)	< 0.0001
Male vs. female	1.05 (0.98–1.12)	0.19
Race		
White	0.88 (0.80–0.98)	0.01
Black or African American	0.92 (0.82–1.04)	0.20
Hispanic or Latino	0.96 (0.81–1.13)	0.62
Overweight and obesity	1.18 (1.01–1.39)	0.04
Malnutrition	1.56 (1.34–1.82)	< 0.0001
Carcinoembryonic Ag (ng/mL)		
0–5	0.97 (0.85–1.09)	0.58
5–10	1.07 (0.90–1.28)	0.45
> 10	1.21 (1.06–1.39)	0.01
Cancer Ag 19‐9 (Units/mL)		
< 37	0.75 (0.66–0.87)	< 0.0001
37–500	0.93 (0.83–1.04)	0.18
500–1000	0.94 (0.79–1.11)	0.44
≥ 1000	1.18 (1.07–1.31)	0.001

As only a portion of the patients had relevant laboratory values available for assessment within the 30‐day window prior to their first documentation of metastatic cancer, further subgroup analyses were performed. The first subgroup analysis included only patients with a documented CA 19‐9 level in the 30‐day window. This yielded 565 patients in the lung cohort and 3098 patients in the liver cohort (Table S3A). Cox regression analysis (Table S3B) revealed that patients presenting with lung metastases experienced better survival as compared to patients in the liver metastases cohort (HR: 0.68, 95% CI: 0.61–0.76, *p* < 0.0001). Normal CA 19‐9 values (≤ 37 U/mL) were associated with improved survival (HR: 0.73, 95% CI: 0.64–0.84, *p* < 0.0001), while high levels of CA 19‐9 (≥ 1000 U/mL) were associated with worse overall survival (HR: 1.2, 95% CI: 1.08–1.34, *p* = 0.0008). A second analysis further focused on patients with documented chemotherapy treatment following their metastatic diagnosis. This yielded 46 patients in the lung cohort and 1982 patients in the liver cohort (Table S4A). Cox regression analysis (Table S4B) again showed that patients presenting with lung metastases experienced better survival as compared to patients in the liver metastases cohort (HR: 0.67, 95% CI: 0.47–0.95, *p* = 0.0234).

## 4 Discussion

Pancreatic ductal adenocarcinoma (PDAC) remains one of the deadliest malignancies, with a 5‐year survival rate of approximately 3% for patients diagnosed with metastatic (stage IV) disease [3]. The liver is the most common site of metastatic spread, followed by the lungs and intraperitoneal dissemination [21]. Prior studies in other cancers, including colorectal cancer, have demonstrated that the site of initial metastasis can influence overall survival [11, 13]. Specifically, patients first presenting with lung metastases often exhibit better outcomes than those presenting with liver metastases [13]. This study sought to determine whether this survival pattern also applies to patients with metastatic PDAC.

Leveraging the strength of the TriNetX global health research network, we conducted a large retrospective cohort study of mPDAC patients who initially presented with either liver only or lung only metastases. The size and diversity of this cohort enhance the robustness and generalizability of our findings. After identifying significant differences in baseline demographics and clinical variables between the two groups, we employed two separate statistical approaches to address the differences: we used propensity score matching to create two well‐balanced cohorts, and we used Cox regression modeling. This matching approach controlled for known confounders such as age, race, nutritional status, CEA, and CA 19‐9 levels.

Our results show that metastatic PDAC patients presenting with lung metastases had a significantly longer median overall survival as compared to those presenting with liver metastases. Most notably, the multivariable Cox proportional hazards model demonstrated that the initial lung metastases cohort was independently associated with an approximate 40% decrease in the risk of death compared to patients presenting with liver metastases, even after adjusting for other prognostic variables.

In addition to the metastatic site, the Cox regression analysis identified several other independent predictors of mortality. Both malnutrition and obesity were associated with increased risk of death, consistent with prior literature linking extremes in weight to worse oncologic outcomes [22]. CA 19‐9, a tumor biomarker commonly used in monitoring and managing PDAC, also showed prognostic value: patients with significantly elevated CA 19‐9 levels had increased mortality risk, while those with normal levels had a significantly reduced risk of death, supporting its role as a surrogate marker of tumor burden [23]. As CA 19‐9 values were not available to all patients, we performed another subgroup analysis to include only patients with documented CA 19‐9 values that were measured within a month prior to diagnosis. Similar to the main Cox regression, this subgroup analysis showed the same significant findings. CEA, another tumor marker commonly used in the oncologic monitoring of PDAC, was also found to have prognostic value, and elevated levels were associated with increased risk of death [23].

Comparison of our findings with previously reported literature further supports our findings (Table 4). Owerira et al. conducted a large National Cancer Institute's Surveillance, Epidemiology, and End Results (SEER)‐based study, identifying a survival advantage in PDAC patients with initial lung versus liver metastases [24]. They have also shown that a small subpopulation (~1%) of patients, who were able to undergo a to surgical resection of their primary or metastatic tumor, showed a markedly improved survival [24]. Our findings build upon these previous findings and take into additional account surrogate markers for metastatic disease extent such as nutritional status, CEA and CA 19‐9 levels, in the context of a multi‐national research network. We found that patients with liver metastases exhibited higher biomarker levels. Both our Cox regression analysis and propensity score matched survival analysis reveal that metastatic PDAC initially presenting in the lungs is independently and strongly associated with improved survival, irrespective of biomarker status. Decoster et al. reported similar survival differences in a smaller case–control study (*n* = 37), reinforcing the association between lung metastases and longer survival [14]. They also observed a higher proportion of women in the lung metastasis group, a finding echoed in our unmatched data, although gender was not a significant predictor in our Cox model.

**TABLE 4:** Comparison of the current study findings to prior research conducted on PDAC patients with lung and liver metastases.

Authors	Study design	Key findings
Oweira et al. [24]	Unmatched retrospective cohort study (*n* = 13,233)	Lung metastases associated with improved survival compared to liver metastases (*p* < 0.0001).
Wu et al. [25]	Retrospective unmatched study (clinical + hospital data)	Isolated lung metastases had longer median survival than liver metastases in both data sets (*p* = 0.008, *p* = 0.044).
Ebrahimi et al. [15]	Retrospective review (*n* = 205)	Statistically significant survival benefit in patients with isolated lung metastases.
Deeb et al. [26]	Case report (*n* = 5)	Patients with lung metastases exhibited longer than expected survival.
Decoster et al. [14]	Retrospective case–control (*n* = 37)	Median survival significantly longer in lung metastases group (20 vs. 9 months, *p* < 0.001). The majority of the lung cohort were women (*p* < 0.001).
Cham et al. [27]	Retrospective cohort study (*n* = 68)	No statistically significant difference in survival (*p* = 0.311), though Kaplan–Meier suggested a trend toward improved survival in the lung metastasis group.
Levi et al. [28]	Retrospective cohort study (*n* = 831)	Median overall survival was significantly longer in the initial lung cohort as compared to the initial liver cohort (*p* < 0.001). Survival advantage for initial lung cohort persisted even with standard of care chemotherapy treatment.
Mancheski et al.	Retrospective cohort study (*n* = 6256; *n* = 848 matched pairs)	Patients presenting with initial lung metastases had better overall survival as compared to patients presenting with initial liver metastases (*p* < 0.001). Increased age, obesity, malnutrition, and increased biomarkers were also found to be associated with worse survival outcomes.

A recent study by Levi et al. investigated survival differences between patients with initial lung and initial liver mPDAC using a commercial U.S.‐based database containing real‐world clinical and molecular data (Perthera Inc., McLean, VA) [28]. Analyzing a total of 831 patients, they found that individuals with initial lung metastases had improved survival compared to those with initial liver metastases, consistent with our findings [28]. Additionally, they found that the survival advantage persisted regardless of type of standard‐of‐care chemotherapy.

Other studies, such as those by Wu et al., Ebrahimi et al., and Deeb et al., support the trend that PDAC patients presenting with lung metastases tend to have longer survival [15, 25, 26]. In contrast, Cham et al. did not find a significant survival difference [27]; however, their study was limited by a very small number of patients with isolated lung metastases (*n* = 7), potentially limiting their ability to detect a statistically significant effect [25].

Yachida et al. examined metastatic and primary tumors from 7 PDAC patients and showed that some tumor heterogeneity exists between metastatic and primary PDAC sites [29]. However, it appears that driver mutations remain unchanged between the primary and metastatic sites [30]. Makohon‐Moore et al. carried out whole genome sequencing of 26 metastases from four PDAC patients. They identified KRAS mutations in every sample of all four patients. Mutations in other driver genes, TP53 and SMAD4, for example were also identified in all samples of each patient [30]. They found that driver genes were highly similar across the founder cells that gave rise to metastases [30].

Currently, researchers are actively searching for the biological mechanism to explain the different prognoses between metastatic sites. One possible explanation of the differences in survival between PDAC patients with lung or liver metastases is the uniqueness of the metastatic site's tumor microenvironment. Ho et al. found that the tumor microenvironment (TME) differs between lung and liver metastases [16]. Briefly, lung metastases had more immune infiltration, activation, and pro‐immune signaling pathways, while, in contrast, liver metastases showed activation of immune‐suppressive pathways. More specifically, the lung showed a great density of CD4+ and CD8+ T cells involved in adaptive immune activation and response [16].

In a recent study, Rathore et al. demonstrated that liver endothelial cells can activate HER3 receptors on PDAC cells [31]. Activation of HER3 receptors can lead to downstream activation of the AKT and MAPK kinase pathways, which are pro‐survival, suggesting this pathway may play a vital role in the survival and proliferation of liver metastases.

Further, a study by Miarka et al. also suggests that a specific tumor necrosis factor (TNF) receptor found in the liver may play a role in promoting cell survival [32]. After knocking down tumor necrosis factor receptor 2 (TRAIL‐R2) expression in a pancreatic cancer cell line, the authors observed fewer macroscopic liver metastases compared to wildtype PDAC cells, suggesting that TRAIL‐R2 is involved in tumor cell proliferation. This finding suggests that PDAC cells expressing TRAIL‐R2 are better able to escape growth control and may play a key role in the growth of liver metastases.

Liver parenchymal hepatocytes may also contribute to creating an environment that supports metastatic cells. In a colorectal cancer study, it was found that binding of cancer cells to hepatocyte‐derived extracellular matrix increased expression of genes that promote cell survival, motility, and proliferation [33].

Another possible mechanism in play is hypothesized to be through formation of premetastatic niches [34]. In general, a premetastatic niche refers to a distant organ that is readied and matured for metastatic implantation of circulating cancer cells due to factors released by the primary tumor. For example, macrophage migration inhibitory factor (MIF) was shown to be an essential component of this premetastatic niche formation, as Stage I PDAC patients who went on to develop liver metastases showed higher levels of MIF in primary tumor‐derived exosomes [34].

Despite its strengths, this study has several limitations inherent to retrospective analyses of this type. It remains unclear if the more favorable prognosis of mPDAC lung metastases stems from a feature inherent to the molecular landscape of the cancer itself or if this less aggressive phenotype stems from the lung microenvironment as we did not assess molecular or immune cell characteristics. Naturally, our analyses can only suggest associations and not prove clear causation. Reliance on structured ICD coding in TriNetX may introduce misclassification or selection bias and limits the assessment of the co‐morbidities in the cohorts to ICD‐10 coded features. For example, the true proportion of patients with obesity or malnutrition in our cohorts is likely not fully captured by the diagnosis codes used for obesity and malnutrition. It is probable that many of the patients in our cohort who are overweight or obese were never specifically assigned such a diagnosis in their electronic medical record. Therefore, it is more likely in the case of malnutrition, overweight or obesity, that the rate of diagnoses is low rather than the true prevalence. This also suggests that both groups are matched to have similar numbers of patients who have ICD codes of obesity or malnourishment, but we have no clear information of the true rates of these conditions. Another limitation of relying on diagnosis codes is the lack of a specific code for metastatic PDAC to the liver or lung. To address that, we identified our patients by using the codes for secondary neoplasm for the lung or the liver occurring with the annotated code of PDAC. To help decrease the risk of including patients without metastatic pancreatic cancer, we only included patients who were diagnosed with liver or lung metastasis after they were diagnosed with PDAC. While this decreases the risk of including patients with other forms of metastatic cancer, it also introduces the possibility of missing patients who received a diagnosis of liver or lung metastases while awaiting a diagnosis of primary PDAC. Another limitation of this study is that we did not account for different treatment types that patients may have received or their baseline functional status that can impact their ability to tolerate chemotherapeutic treatments. Unfortunately, we have found that chemotherapy data was not well captured in this patient population, resulting in a very small cohort of patients with lung metastases (*N* = 46) and preventing any higher‐level assessment of differential responses to various therapies. However, within the chemotherapy‐treated population of patients, we have noted the metastatic site to carry the same effect size as for the overall cohort. Additionally, we were unable to reliably capture whether mPDAC patients in our cohorts underwent any surgical resection of their primary or metastatic disease. However, these risk factors are not available usually a priori upon, but are revealed upon assessment of tumor biology and response to therapy.

## 5 Conclusions

Metastatic PDAC remains a devastating diagnosis and can be difficult to treat. Our large retrospective cohort study shows and validates that the initial presentation with lung metastases is associated with better overall survival as compared to the initial presentation with liver metastases in this patient group. This furthermore suggests that providers can use this observation for prognostic stratification to help patients and families make more informed decisions regarding aggressiveness of treatments, quality‐of‐life goals, and possible end‐of‐life planning. The biological mechanisms behind the difference in survival remain unclear, and future research should aim to understand which factors drive the difference in survival and what type of treatments should be employed to maximize patient survival and quality of life.

## Author Contributions

**Eleanor Mancheski:** methodology, investigation, formal analysis, writing (original draft preparation, review and editing). **David Baek:** methodology, investigation, formal analysis, writing (original draft preparation, review and editing). **Wilbur Bowne:** conceptualization, Writing (review and editing). **Harish Lavu:** conceptualization, Writing (review and editing). **Charles J. Yeo:** conceptualization, Writing (review and editing). **Avinoam Nevler:** conceptualization, methodology, investigation, supervision, writing (original draft preparation, review, and editing).

## Funding

The authors have nothing to report.

## Conflicts of Interest

The authors declare no conflicts of interest.

## Supporting information


**Data S1:** cam471760‐sup‐0001‐Tables.docx.

## Data Availability

The data that support the findings of this study are available from TriNetX. Restrictions apply to the availability of these data, which were used under license for this study. Data are available from the author(s) with the permission of TriNetX.
