# Supplementary material for: Initial Site of Metastasis Influences Prognosis in Pancreatic Ductal Adenocarcinoma
Source: Cancer Med. 2026 Mar 26;15(4):e71760. doi: 10.1002/cam4.71760 (PMC13140831; doi:10.1002/cam4.71760)
Supplement: Supplementary file 1 — Data S1: cam471760‐sup‐0001‐Tables.docx. [file CAM4-15-e71760-s001.docx]

**SUPPLEMENTARY**

**Supplementary Table 1.** ICD-10-CM codes used to generate the PDAC with initial liver metastases cohort.

| ICD-10-CM Code | Code Name |
| --- | --- |
| CD25.0 | Malignant neoplasm of head of pancreases |
| CD25.1 | Malignant neoplasm of body of pancreas |
| CD25.2 | Malignant neoplasm of the tail of pancreas |
| CD25.3 | Malignant neoplasm of pancreatic duct |
| CD25.7 | Malignant neoplasm of other parts of pancreas |
| CD25.8 | Malignant neoplasm of overlapping sites of pancreas |
| CD25.9 | Malignant neoplasm of pancreas, unspecified |
| C78.7 | Secondary malignant neoplasm of liver and intrahepatic bile duct |

**Supplementary Table 2.** ICD-10-CM codes used to generate the PDAC with initial lung metastases cohort.

| ICD-10-CM Code | Code Name |
| --- | --- |
| C25.0 | Malignant neoplasm of head of pancreases |
| C25.1 | Malignant neoplasm of body of pancreas |
| C25.2 | Malignant neoplasm of the tail of pancreas |
| C25.3 | Malignant neoplasm of pancreatic duct |
| C25.4 | Malignant neoplasm of endocrine pancreas |
| C25.7 | Malignant neoplasm of other parts of pancreas |
| C25.8 | Malignant neoplasm of overlapping sites of pancreas |
| C25.9 | Malignant neoplasm of pancreas, unspecified |
| C78.0 | Secondary malignant neoplasm of lung |

**Supplementary Table 3A.** Demographic Characteristics. Subgroup analysis limited to patients with available CA19-9 measurements.

|  | **Initial Lung Metastasis** | **Initial Liver Metastasis** |
| --- | --- | --- |
| N | 565 | 3098 |
| Age | 76±9 | 74±10 |
| Male | 255 (45.1%) | 1662 (53.7%) |
| Female | 310 (54.9%) | 1436 (46.4%) |
| White | 449 (79.5%) | 2370 (76.5%) |
| Black | 66 (11.7%) | 495 (16%) |
| Asian | 21 (3.7%) | 94 (3%) |
| Other | 29 (5.2%) | 139 (4.5%) |
| Overweight & Obesity | 113 (20%) | 576 (18.6%) |

**Supplementary Table 3B.** Cox multi-hazard regression model of overall survival. Subgroup analysis limited to patients with available CA19-9 measurements.

| **Covariate** | **Hazard Ratio (95% CI)** | **P > \|z\|** |
| --- | --- | --- |
| **Lung mets. vs. Liver mets.** | 0.68 (0.606, 0.763) | <0.0001 |
| **Age at Index (risk/year)** | 1.01 (1.006, 1.015) | <0.0001 |
| **Male vs. Female** | 1.069 (0.987, 1.158) | 0.103 |
| **Race/Ethnicity** |  |  |
| White | 0.934 (0.798, 1.093) | 0.395 |
| Black or African American | 1.021 (0.849, 1.229) | 0.823 |
| Hispanic or Latino | 1.197 (0.947, 1.512) | 0.133 |
| **Overweight and obesity** | 1.006 (0.815, 1.241) | 0.959 |
| **Malnutrition** | 1.666 (1.403, 1.977) | <0.0001 |
| **Carcinoembryonic Ag [ng/mL]** |  |  |
| <37 | 0.734 (0.638, 0.844) | <0.0001 |
| 37 - 500 | 0.925 (0.826, 1.036) | 0.177 |
| 500 - 1000 | 0.969 (0.820, 1.144) | 0.708 |
| ≥1000 | 1.203 (1.080, 1.340) | 0.001 |
| **Cancer Ag 19-9 [Units/mL]** |  |  |
| 0 - 5 | 0.961 (0.841, 1.097) | 0.552 |
| 5 - 10 | 0.981 (0.819, 1.175) | 0.832 |
| >10 | 1.21 (1.055, 1.388) | 0.006 |

**Supplementary Table 4A.** Demographic Characteristics. Subgroup analysis limited to patients with available CA19-9 measurements and chemotherapy treatment after diagnosis of metastatic pancreatic ductal adenocarcinoma.

|  | **Initial Lung Metastasis** | **Initial Liver Metastasis** |
| --- | --- | --- |
| N | 46 | 1982 |
| Age | 73±8 | 73±10 |
| Male | 26 (56.5%) | 1058 (53.4%) |
| Female | 20 (43.5%) | 920 (46.4%) |
| White | 34 (73.9%) | 1538 (77.6%) |
| Black | 10 (21.7%) | 309 (15.6%) |
| Asian | 0 (0%) | 56 (2.8%) |
| Other | 2 (4.4%) | 79 (4%) |
| Overweight & Obesity | 13 (28.3%) | 412 (20.8%) |

**Supplementary Table 4B.** Cox multi-hazard regression model of overall survival. Subgroup analysis limited to patients with available CA19-9 measurements and chemotherapy treatment after diagnosis of metastatic pancreatic ductal adenocarcinoma.

| **Covariate** | **Hazard Ratio (95% CI)** | **P > \|z\|** |
| --- | --- | --- |
| **Lung mets. vs. Liver mets.** | 0.669 (0.473, 0.947) | 0.023 |
| **Age at Index (risk/year)** | 1.006 (1.001, 1.012) | 0.018 |
| **Male vs. Female** | 1.104 (0.995, 1.226) | 0.063 |
| **Race/Ethnicity** | 0.89 (0.718, 1.103) | 0.287 |
| White | 0.932 (0.727, 1.196) | 0.581 |
| Black or African American | 1.101 (0.816, 1.485) | 0.528 |
| Hispanic or Latino | 1.168 (0.906, 1.507) | 0.23 |
| **Overweight and obesity** | 1.764 (1.394, 2.232) | 0 |
| **Malnutrition** |  |  |
| **Carcinoembryonic Ag [ng/mL]** | 0.731 (0.612, 0.874) | 0.001 |
| <37 | 0.869 (0.750, 1.007) | 0.061 |
| 37 - 500 | 0.973 (0.795, 1.191) | 0.792 |
| 500 - 1000 | 1.171 (1.022, 1.342) | 0.023 |
| ≥1000 |  |  |
| **Cancer Ag 19-9 [Units/mL]** | 0.951 (0.800, 1.132) | 0.574 |
| 0 - 5 | 0.973 (0.770, 1.228) | 0.817 |
| 5 - 10 | 1.113 (0.937, 1.321) | 0.224 |
| >10 | 0.669 (0.473, 0.947) | 0.023 |
